# Diagnostic Significance of Serum Long Noncoding HOX Antisense Intergenic Ribonucleic Acid in Patients with Hepatitis B Virus Related Hepatocellular Carcinoma

**DOI:** 10.5152/tjg.2024.23314

**Published:** 2024-05-01

**Authors:** Yue Pan, Meng Cai, Fan Zhang, Xiaoming Liu, Menghua Li, Bingxin Xie, Ju Li

**Affiliations:** Department of Clinical Laboratory, The Public Health Clinical Center of Dalian, Dalian, Liaoning Province, China

**Keywords:** Alpha-fetoprotein, hepatocellular carcinoma, HOX transcript antisense RNA, long noncoding RNAs, serum biomarker

## Abstract

**Background/Aims:**

Hepatocellular carcinoma (HCC) is one of the most common malignant tumors and the third leading cause of cancer-related mortality. Extensive literature suggests that long noncoding RNAs play a role in the progression of HCC and hold potential as diagnostic biomarkers for this disease.

**Materials and Methods:**

We examined the serum levels of HOX antisense intergenic RNA (HOTAIR) in 49 hepatitis patients, 31 liver cirrhosis (LC), and 37 HCC patients using quantitative real-time polymerase chain reaction. Correlations between serum HOTAIR levels and clinical data were evaluated in HCC patients. The receiver operating characteristic curve was utilized to analyze the diagnostic potency of HOTAIR.

**Results:**

The HOTAIR levels in serum were significantly higher in HCC patients compared to those with hepatitis (*P* = .003) and LC patients (*P* = .048). There was a significant association between the serum levels of HOTAIR and positivity of hepatitis B e antigen (HBeAg) (*P* = .039) as well as portal vein tumor thrombus (*P* = .040) in HCC patients. The area under the curve (AUC) for HOTAIR for distinguishing HCC from hepatitis and LC was 0.697. The combined AUC for HOTAIR, HBeAg, and alpha-fetoprotein (AFP) was 0.777.

**Conclusion:**

Serum HOTAIR functions as a potential diagnostic marker for hepatitis B virus-related HCC. Combining HOTAIR with clinical data and AFP can reinforce the diagnostic precision on HCC.

**Main Points**

- We found that serum HOX antisense intergenic RNA (HOTAIR) levels in hepatocellular carcinoma (HCC) were significantly higher than those in hepatitis and liver cirrhosis patients.
- Serum HOTAIR levels in HCC patients were associated with portal vein tumor thrombus and hepatitis B e antigen (HBeAg) positivity.
- HOX antisense intergenic RNA may be a potential marker for diagnosing hepatitis B virus-related HCC.
- The combination of HOTAIR, alpha-fetoprotein, and HBeAg improves the diagnostic efficacy of HCC.

## Introduction

Hepatocellular carcinoma (HCC) is the sixth most common cancer worldwide and a major type of primary liver cancer.^1-3^ The HCC has a higher incidence in developing regions, including China, the Republic of Korea, and sub-Saharan Africa.^4^ Infection with the hepatitis B virus remains a contributing factor for HCC.^5,6^ A previous study demonstrated that the incidence rates for HCC among cirrhosis ranged from 1% to 8% per year.^3^ However, traditional biomarkers have limitations in diagnosing early-stage HCC. Despite being widely used for HCC diagnosis, serum alpha-fetoprotein (AFP) has been criticized due to its low sensitivity and specificity.^7^ It has been noted that more than 30% of early-stage HCC patients with small tumors showed the same AFP levels as those in healthy control;^8^ moreover, serum AFP levels can even be within normal ranges in 15%-30% of advanced HCC.^9^ The low sensitivity of AFP is the main drawback for HCC diagnosis. Thus, better biomarkers that could predict early HCC and HCC progression are an urgent need.

Long noncoding RNAs (lncRNAs) are a class of non-protein-coding RNAs that participate in cellular growth, proliferation, and invasion.^10,11^ Emerging evidence suggests that lncRNAs show differential expression in cancer progression and play crucial roles in tumorigenesis.^12,13^ As a well-studied lncRNA, HOTAIR is known as an oncogene and demonstrates elevated expression in various cancers such as glioblastoma, lung cancer, and cervical cancer.^14-16^ HOX antisense intergenic RNA is associated with epigenetic regulation, cancer susceptibility, poor survival, tumor recurrence, and immune escape.^17,18^ Furthermore, it has also been proven that HOTAIR levels were higher in HCC tissues compared to normal liver tissues.^19^ Additionally, the transcriptional activity of HOTAIR was positively correlated with poor prognosis and disease progression in HCC.^20^ An earlier report also indicated that HOTAIR served as a predictive factor for HCC recurrence in liver transplantation patients.^19^ Owing to these findings, we hypothesize that HOTAIR may be a potential indicator for HCC.

Based on the findings of previous studies, our study tries to (i) analyze the serum HOTAIR level in hepatitis B virus (HBV)-related hepatitis, liver cirrhosis (LC), and HCC patients, (ii) find out the relationship between HOTAIR and clinical data in HCC patients, and (iii) explore the accuracy of serum HOTAIR in HCC patients and diagnostic significance of serum HOTAIR.

## Materials and Methods

### Study Group

This study enrolled 49 patients with HBV infection, 31 patients diagnosed with LC and 37 individuals diagnosed with HCC between December 2021 and March 2023 from the Public Health Clinical Center of Dalian. All participants were infected with HBV and received antiviral drugs such as tenofovir or entecavir. Patients who had a history of chemotherapy or radiotherapy and those who were infected with HCV were ruled out of the study.

All of the patients’ data collected were analyzed without identifiable personal information. Written informed consent was obtained from the patients included in the study. The study was approved by the Ethical Committee of The Public Health Clinical Center of Dalian (Approval number: 2019-026-003).

### Blood Samples and Laboratory Testing

Peripheral venous blood was collected from each participant. All blood samples were tested immediately after being collected from participants. Alanine aminotransferase (ALT), aspartate aminotransferase (AST), alkaline phosphatase (ALP), γ-glutamyl transpeptidase (γ-GT), and total bilirubin (TBIL) were tested by an automated chemistry analyzer (ADVIA 2400, SIEMENS AG, Germany). Hepatitis B surface antigen (HBsAg) and antibody (HBsAb), hepatitis B e antigen (HBeAg), and antibody (HBeAb) and HBV core antigen (HBcAb) were tested by chemiluminescence analyzer (Architect i2000SR, Architect, USA), according to the manufacturer’s recommendations. HBV DNA was detected by a Roche Light Cycler 480 real-time fluorescence quantitative PCR instrument. AFP was detected by the Cobas e411 module (Roche Diagnostics, Germany).

### RNA Extraction and Quantitative Real-Time Polymerase Chain Reaction

LncRNAs were detected by the lnRcute lncRNA qPCR Kit (TIANGEN, Beijing, China). RNA was extracted from 200 µL serum by the RNA isolation commercial kit (TIANGEN, Beijing, China) following its instructions. Reverse transcription of RNA was carried out with lnRcute lncRNA First-Strand cDNA kit (TIANGEN, Beijing, China) by step. The reference gene *GADPH* was used for normalization of the expression levels. The lncRNA HOTAIR primer sequence is F: 5’-GGTAGAAAAAGCAACCACGAAGC-3’, R: 5’-ACATAAACCTCTGTCTGTGAGTGCC-3’. The GAPDH primer sequence is F: 5’-CATCTTCTTTTGCGTCGCCA-3’, R: 5’-TTAAAAGCAGCCCTGGTGACC-3’. Fold changes were calculated using 2-ΔΔCt for relative quantification.

### Statistical Analysis

The Statistical Package for the Social Sciences Statistics software, version 23.0 (IBM Inc., Armonk, NY, USA), was applied to the analyzed data. Normally distributed variables are presented as mean ± SD, while nonparametric variables are presented as median and interquartile ranges. Mann–Whitney *U*-test and Kruskal–Wallis *H*-test were conducted to compare between any 2 independent groups. Pearson’s chi-square *χ*2 test was used to compare qualitative variables among the HCC and LC hepatitis groups. The receiver operating characteristic (ROC) analysis was performed to estimate the diagnostic value and assess the area under curve (AUC), cutoff values, sensitivity and specificity. A *P* < .05 was considered statistically significant.

## Results

### Demographic and Laboratory Data of the Study Group

The demographic and biochemical data of patients, including serum AST, ALT, PA, γ-GT, ALP, TBIL, DBIL, HBsAg, HBsAb, HBeAg, HBeAb, and HBV DNA levels, are presented in Table 1. There were no differences in gender or age among the hepatitis, LC, and HCC groups. The phase of hepatitis B is shown in Supplementary Table 1, and the expression of HOTAIR did not vary across the groups. The clinical stage of cirrhosis patients is presented in Supplementary Table 2, and no significant differences were observed in HOTAIR expression between the 2 groups. It should be noted that all patients with HCC had cirrhosis.

### Serum HOX Antisense Intergenic Ribonucleic Acid Levels in Hepatocellular Carcinoma, Liver Cirrhosis, and Hepatitis Patients

**Figure 1.**
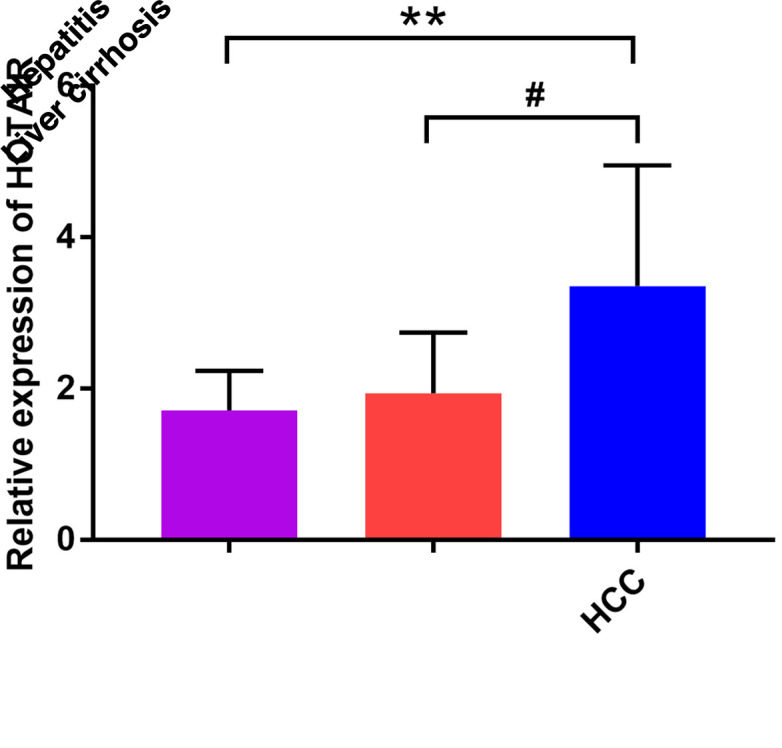
The expression of HOTAIR in serum.

Serum HOTAIR levels were higher in HCC patients compared to hepatitis (*P* = .003) and LC patients (*P* = .048). No significant difference in the serum HOTAIR levels was shown between LC and hepatitis patients (Figure 1).

### Association Between Serum HOX Antisense Intergenic Ribonucleic Acid and Clinical Data in Hepatocellular Carcinoma

The correlation between serum HOTAIR levels and clinical data of HCC patients is displayed in Table 2. The results revealed a close correlation between serum HOTAIR levels and HBeAg (*P* = .039) positivity in HCC patients. Serum HOTAIR levels in HBeAg-negative HCC patients were higher than that observed in HBeAg-positive HCC patients. Moreover, serum HOTAIR level was significantly correlated with PVTT (*P* = .040). However, no significant correlation was found between HOTAIR levels and AST, ALT, AFP, presence of extrahepatic metastasis, and size or number of HCC lesions.

### Diagnostic Accuracy of Serum HOX Antisense Intergenic Ribonucleic Acid for Hepatocellular Carcinoma

We utilized ROC curve analysis to assess the sensitivity, specificity, and diagnostic accuracy of HOTAIR for HCC patients. Additionally, we individually examined the diagnostic value of AFP and HBeAg, as well as their combination with the HOTAIR, for diagnosing HCC (Table 3).

At a cutoff value of 1.32, the AUC of HOTAIR for distinguishing the HCC group from the hepatitis and LC groups was 0.697, with a sensitivity of 78% and a specificity of 65%. We also evaluated the diagnostic value of AFP for HCC, which yielded an AUC of 0.726 with a sensitivity of 43% and a specificity of 100% at a cutoff level of 63.81, indicating poor sensitivity for diagnosing HCC.

We found that the combination of AFP, HOTAIR, and the reciprocal index of HBeAg (1/HBeAg) had a high AUC of 0.829 when distinguishing HCC from the hepatitis group. The AUC was 0.777 when AFP, HOTAIR, and 1/HBeAg were combined to distinguish HCC from LC and the hepatitis group. This finding suggests that the combination of HOTAIR, AFP, and 1/HBeAg has a stronger diagnostic efficiency compared to using only HOTAIR or AFP alone.

## Discussion

In the present study, we found that HOTAIR may be a useful tumor marker for diagnosing HBV-related HCC. Combining HOTAIR with clinical data and AFP improves the diagnostic efficacy of HCC. Increasing evidence proves that HOTAIR acted as an oncogene and took part in the initiation and progression of HCC.^21,22^ A lot of basic research has explored and revealed the foundational mechanism of HOTAIR in HCC development and progression.^23,24^ Based on clinical research, previous studies reported that the expression of HOTAIR in HCC tissues was higher than in adjacent noncancerous tissues.^19,25^ Recently, it has become a noninvasive and convenient way to detect serum proteins, lncRNA, and other molecules for disease diagnosis.^26,27^ In our study, we demonstrated that the expression of serum HOTAIR was obviously elevated in HBV-related HCC patients compared to LC and hepatitis groups. This result is in line with the findings of Lou et al.^28^ They demonstrated that, compared to liver cirrhosis and healthy groups, the serum level of HOTAIR was higher in HCC patients.

We tried to explore the association between serum HOTAIR levels and clinical data in HCC patients. We found that serum HOTAIR levels were correlated with PVTT. The previous paper also reported that the HOTAIR level in HCC tissues was correlated to tumor node metastasis, extrahepatic metastasis, vascular invasion, and tumor size.^25^ High expression of HOTAIR in HCC tissues indicated a significantly short recurrence-free survival.^19^ In assistance with this data, we considered that HOTAIR might be a suitable predictive biomarker for HCC diagnosis and prognosis. In addition, we revealed that the levels of HOTAIR were significantly correlated with HBeAg positivity. Recent findings have shown that hepatocellular carcinoma can still develop in a substantial proportion of HBeAg-negative patients.^29,30^ Consistent with these findings, we discovered that 23 out of 37 participants with HCC (62.2%) were HBeAg-negative (HBeAg <1.0 S/CO).

To assess the diagnostic value of HOTAIR, ROC analysis was conducted, which held an AUC of 0.697 with a sensitivity of 78% in distinguishing HCC from hepatitis and LC groups. We speculated that HOTAIR could be a promising tumor marker for detecting HCC. Interestingly, our analysis revealed that 1/HBeAg held 95% sensitivity in discriminating HCC from hepatitis and LC groups. We attempted to incorporate the value of HBeAg into a combined diagnosis and found that when using both markers together with AFP, the AUC was improved to 0.777 for diagnosing HCC accurately. Therefore, combing these markers together can improve the diagnostic accuracy of HCC.

The study had several limitations. First, only 37 HCC patients were enrolled in the research, and we did not include healthy individuals in the control groups. Secondly, although all participants used antiviral drugs, some patients took both tenofovir and entecavir. The impact of different types of drugs on HOTAIR expression is also unknown. Thirdly, we also did not know the effect of fatty liver on the outcome. All participants were from China; therefore, the results may not apply to patients from other racial groups. The novelty of this study was that all participants were hepatitis B-related patients, and all HCC patients had cirrhosis.

In conclusion, we validated that the expression of serum HOTAIR is higher in HCC patients compared to hepatitis and LC patients. Additionally, a significant correlation exists between the serum levels of HOTAIR and HBeAg, as well as PVTT, in HCC patients. Furthermore, HOTAIR has the potential to be a helpful diagnostic marker for distinguishing HCC from hepatitis and LC groups. The combination of HOTAIR and clinical tests with AFP further improves the diagnostic accuracy of HCC. We plan to confirm the diagnostic accuracy of serum HOTAIR in a larger cohort of HCC patients in the near future.

## Figures and Tables

**Table 1.** Demographic and Laboratory Data of the Patients

Data/Characteristics	Hepatitis	Liver Cirrhosis	HCC
Number	49	31	37
Age (years)	53.27 ± 9.91	56.23 ± 10.82	57.35 ± 7.74
Male/female	27/22	17/14	20/17
AST (U/L)	21.30 (14.00-37.65)	39.80 (24.20-60.70)	28.50 (20.45-46.60)
ALT (U/L)	21.90 (17.80-43.15)	32.20 (19.80-112.90)	23.60 (16.80-47.75)
PA (mg/L)	216.20 ± 64.28^**^	136.70 ± 63.48	128.40 ± 77.02
γ-GT (U/L)	40.70 (18.15-72.45)^*^	67.10 (51.70-98.30)	65.75 (28.93-106.00)
ALP (U/L)	59.50 (41.25-74.85)	53.40 (24.60-146.80)	71.40 (51.13-108.90)
TBIL (µmol/L)	15.90 (10.60-22.50)^*^	22.40 (11.50-39.40)	19.90 (13.25-33.20)
DBIL (µmol/L)	4.70 (3.50-7.15)^**^	8.60 (5.30-13.80)	8.85 (4.70-14.95)
HBsAg (IU/mL)	>250.00^*^	250.00 (84.00-250.00)	250.00 (56.56-250.00)
HBsAb (mIU/mL)	0.72 (0.29-1.45)	1.02 (0.34-2.02)	0.63 (0.25-1.29)
HBeAg (N/P)	17/32^*^	18/13	23/14
HBeAb (N/P)	14/35	14/17	16/21
HBV-DNA (IU/mL) <300/≥300	19/30^***^	18/13^###^	32/5

No specific value is displayed when the HBsAg value is greater than 250 S/CO.

AFP, alpha-fetoprotein; ALP, alkaline phosphatase; ALT, alanine aminotransferase; AST, aspartate aminotransferase; DBIL, direct bilirubin; γ-GT, γ-glutamyl transferase; HBcAb, HBV core antigen; HBeAb, hepatitis B antibody; HBeAg, hepatitis B e antigen; HBsAb, hepatitis B surface antibody; HBsAg, hepatitis B surface antigen; HCC, hepatocellular carcinoma; LC, liver cirrhosis; PA, prealbumin; TBIL, total bilirubin; N, negative; P, positive.

*P < .05, HCC vs. hepatitis.

**P < .01.

***P < .001.

^###^ *P* < .001, HCC vs. LC.

**Table 2.** Relationship Between Serum LncRNA-HOTAIR Levels and Clinical Data in Hepatocellular Carcinoma Patients

Characteristics	n	HOTAIR Relative Expression	*P*
Gender			.267
Male	21	1.59 (1.01, 3.21)	
Female	16	2.12 (1.67, 5.94)	
Age			.212
≤55	13	1.59 (0.9, 2.80)	
>55	24	2.29 (1.56, 3.47)	
ALT			.853
≤45	26	2.03 (1.14, 3.39)	
>45	11	1.96 (1.21, 3.09)	
AST			.448
≤45	24	1.75 (1.04, 3.39)	
>45	13	2.29 (1.62, 3.09)	
AFP			.540
≤200	24	1.87 (1.12, 3.27)	
>200	13	2.38 (1.36, 3.32)	
HBeAg			.039
<1	23	2.38 (1.57, 3.61)	
≥1	14	1.58 (0.71, 2.38)	
BCLC stage			.065
0-A	15	1.34 (0.75, 2.92)	
B-D	22	2.29 (1.59, 3.72)	
Extrahepatic metastasis			.326
Yes	6	2.53 (1.53, 7.24)	
No	31	1.75 (0.98, 3.03)	
PVTT			.040
Yes	9	4.05 (1.74, 7.56)	
No	28	1.73 (1.07, 2.82)	
Tumor size			.257
<5 cm	27	2.12 (0.98, 2.98)	
≥5 cm	10	2.01 (1.63, 7.24)	
Tumor number			.245
Single	19	1.75 (0.82, 2.92)	
Multiple	18	2.29 (1.53, 4.22)	

Significant correlation at *P* < .05.

AFP, alpha-fetoprotein; ALT, alanine aminotransferase; AST, aspartate aminotransferase; BCLC, Barcelona Clinic Liver Cancer; HBeAg, hepatitis B e antigen; HCC, hepatocellular carcinoma; LC, liver cirrhosis; PA, prealbumin; PVTT, portal vein tumor thrombus.

**Table 3.** Diagnostic Efficacy of Serum HOTAIR and Clinical Data in Distinguishing Hepatocellular Carcinoma from Hepatitis and LC

Characteristics	AUC	Sensitivity (%)	Specificity (%)	Cutoff	*P*
HCC vs. hepatitis					
HOTAIR	0.701	73	69	1.53	.001
AFP	0.737	73	71	4.51	<.001
1/HBeAg	0.709	81	55	0.22	.002
HOTAIR + 1/HBeAg + AFP	0.829	60	97	–	<.001
HCC vs. LC					
HOTAIR	0.690	78	68	1.29	.007
AFP	0.701	43	100	63.81	.006
1/HBeAg	0.595	92	29	0.09	.172
HOTAIR+1/HBeAg +AFP	0.733	49	100	–	.002
HCC vs. hepatitis + LC					
HOTAIR	0.697	78	65	1.32	.001
AFP	0.726	43	100	63.81	<.001
1/HBeAg	0.664	95	36	0.03	.006
HOTAIR + 1/HBeAg + AFP	0.777	60	90	–	<.001

1/HBeAg, the reciprocal index of HbeAg; AUC, area under the curve; AFP, alpha-fetoprotein; HCC, hepatocellular carcinoma; LC, liver cirrhosis.

**Supplementary Table 1.** Phase of Hepatitis B in Hepatitis B Virus Patients

Phase of Hepatitis B	n	HOTAIR Relative Expression
HBeAg positive chronic infection	23	0.96 (0.45-1.51)
HBeAg positive chronic hepatitis	9	0.77 (0.31-2.41)
HBeAg negative chronic infection	15	2.13 (0.48-4.06)
HBeAg negative chronic hepatitis	2	0.31 (0.18-0.44)

No significant different in HOTAIR levels in four groups. HBsAg, hepatitis B surface antigen; HOTAIR, HOX antisense intergenic RNA.

**Supplementary Table 2.** Clinical Stage of Liver Cirrhosis Patients

Clinical Stage of Cirrhosis	n	HOTAIR Relative Expression
Compensated cirrhosis	5	0.64 (0.37-0.69)
Decompensated cirrhosis	26	1.08 (0.69-2.64)

No significant difference in HOTAIR levels between compensated and decompensated cirrhosis group. HOTAIR, HOX antisense intergenic RNA.
